# Pharmacological intervention to restore connectivity deficits of neuronal networks derived from ASD patient iPSC with a TSC2 mutation

**DOI:** 10.1186/s13229-020-00391-w

**Published:** 2020-10-19

**Authors:** Mouhamed Alsaqati, Vivi M. Heine, Adrian J. Harwood

**Affiliations:** 1 Neuroscience and Mental Health Research Institute, Hadyn Ellis Building, Cathays, Cardiff, CF24 4HQ UK; 2 Division of Psychological Medicine and Clinical Neurosciences (DPMCN), School of Medicine, Cardiff University, Cardiff, UK; 3 School of Bioscience, The Sir Martin Evans Building, Museum Ave, Cardiff, CF10 3AX UK; 4 Department of Complex Trait Genetics, Center for Neurogenomics and Cognitive Research, Amsterdam Neuroscience, Vrije Universiteit Amsterdam, Amsterdam, The Netherlands; 5 Child and Youth Psychiatry, Emma Children’s Hospital, Amsterdam UMC, Amsterdam Neuroscience, Vrije Universiteit Amsterdam, Amsterdam, The Netherlands

## Abstract

**Background:**

Tuberous sclerosis complex (TSC) is a rare genetic multisystemic disorder resulting from autosomal dominant mutations in the *TSC1* or *TSC2* genes. It is characterised by hyperactivation of the mechanistic target of rapamycin complex 1 (mTORC1) pathway and has severe neurodevelopmental and neurological components including autism, intellectual disability and epilepsy. In human and rodent models, loss of the TSC proteins causes neuronal hyperexcitability and synaptic dysfunction, although the consequences of these changes for the developing central nervous system are currently unclear.

**Methods:**

Here we apply multi-electrode array-based assays to study the effects of TSC2 loss on neuronal network activity using autism spectrum disorder (ASD) patient-derived iPSCs. We examine both temporal synchronisation of neuronal bursting and spatial connectivity between electrodes across the network.

**Results:**

We find that ASD patient-derived neurons with a functional loss of *TSC2*, in addition to possessing neuronal hyperactivity, develop a dysfunctional neuronal network with reduced synchronisation of neuronal bursting and lower spatial connectivity. These deficits of network function are associated with elevated expression of genes for inhibitory GABA signalling and glutamate signalling, indicating a potential abnormality of synaptic inhibitory–excitatory signalling. mTORC1 activity functions within a homeostatic triad of protein kinases, mTOR, AMP-dependent protein Kinase 1 (AMPK) and Unc-51 like Autophagy Activating Kinase 1 (ULK1) that orchestrate the interplay of anabolic cell growth and catabolic autophagy while balancing energy and nutrient homeostasis. The mTOR inhibitor rapamycin suppresses neuronal hyperactivity, but does not increase synchronised network activity, whereas activation of AMPK restores some aspects of network activity. In contrast, the ULK1 activator, LYN-1604, increases the network behaviour, shortens the network burst lengths and reduces the number of uncorrelated spikes.

**Limitations:**

Although a robust and consistent phenotype is observed across multiple independent iPSC cultures, the results are based on one patient. There may be more subtle differences between patients with different *TSC2* mutations or differences of polygenic background within their genomes. This may affect the severity of the network deficit or the pharmacological response between TSC2 patients.

**Conclusions:**

Our observations suggest that there is a reduction in the network connectivity of the in vitro neuronal network associated with ASD patients with *TSC2* mutation, which may arise via an excitatory/inhibitory imbalance due to increased GABA-signalling at inhibitory synapses. This abnormality can be effectively suppressed via activation of ULK1.

## Introduction

Tuberous sclerosis complex (TSC) is a developmental genetic disorder characterised by the widespread progression of benign tumours in multiple organs. It affects approximately 1:6000 individuals and is caused by mutations in either *TSC1* or *TSC2* [1]. The most common neurological symptoms associated with TSC are: epilepsy which occurs in 80–90% of patients and is often unmanageable, autism spectrum disorder (ASD) or intellectual disability, which occurs in approximately 50% [1]. The TSC1 and TSC2 proteins form a heterodimer complex that binds to a third subunit (TBC1D7) to form the TSC complex. This complex acts as a GTPase activating protein (GAP). TSC2 protein consists of the GAP domain and most phosphorylation sites, whereas the TSC1 acts as a stabiliser of the complex and prevents TSC2 degradation [2]. Both TSC1 and TSC2 are involved in the stability of the complex and for that reason, patients with either gene mutation present with similar clinical phenotypes. The main role of the TSC complex is the regulation of mechanistic target of rapamycin (mTOR) that exists as two functionally distinct complexes termed, mTOR complex 1 and 2 (mTORC1 and mTORC2) [3]. The mTORC1 pathway plays key roles in multiple cellular processes, such as cell growth and division, autophagy and transcription [4, 5]. Loss of *TSC1* or *TSC2* function activates mTOR signalling, which results in an mTORC1‐dependent increase in ribosomal protein S6 (rpS6), rpS6 kinase 1 (S6K1) eukaryotic initiating factor 4E-binding protein 1 (4E‐BP1) phosphorylation [6, 7]. Aberrant mTORC1 signalling has been identified in cancer progression, diabetes and aging and is involved in increasing cell growth and proliferation [2, 8].

Reports demonstrate that hyperactivation of mTORC1 signalling in neurons is associated with aberrant axonal and dendritic connectivity, enlarged soma size, increased cellular stress, reduced myelination, synaptic dysfunction and neuronal hyperexcitability [9–12]. In animal models, treatment with mTORC1 inhibitor, rapamycin recovered the behavioural deficits including learning, memory and autistic-like features and the neuronal hyperexcitability [13–15]. In human models, rapamycin reversed neuronal hyperexcitability and improved recall memory in patients with angiomyolipomas associated with TSC [16–18]. However, other neuropsychological measures including executive functions and recognition memory showed reduction in some participants [18]. Additionally, rapamycin did not ameliorate neurocognitive dysfunction or behavioural issues in children with TSC [18].

An alternative to direct inhibition of mTOR is to target mTORC1 via AMP-activated protein kinase (AMPK) and Unc-51-like Autophagy Activating Kinase 1 (ULK1), which both inhibit mTORC1 by phosphorylation of its Raptor subunit [19, 20]. Furthermore, AMPK targets mTORC1 indirectly by phosphorylation and activation of ULK1, and activation of TSC1/TSC2 [21, 22]. However, mTORC1 activity inhibits ULK-1 [19, 20], and therefore, in TSC2 deficit cells ULK-1 activity may experience higher levels of inhibition [23, 24]. In principle, activation of either AMPK or ULK1 should alleviate the effect of hyperactivation of mTORC1 in TSC2 deficit cells; however, the outcome of drug treatments that target AMPK or ULK1 will be dependent on the balance of activity of each kinase within the target cells.

In this study, we demonstrate abnormal neuronal network behaviour in patient-derived neurons with a *TSC2* gene mutation and use a novel pharmacological approach to suppress this neuronal dysfunction. We show that although the previously observed neuronal hyperexcitability of TSC patient-derived neurons was suppressed by rapamycin, aberrant neuronal network behaviour could not be rescued. Of the two protein kinases, AMPK and ULK1, only ULK1 activation reduced all aspects of the patient cell phenotype. This strategy of suppressing the disease state could be further developed as a new treatment for TSC patients.

## Materials and methods

### iPSCs and neuronal cultures

TSC patient iPSC clone hVS-417 (P2C) from patient 2 was obtained from the Coriell Biorepository (all teenage donors), and two independent iPSC clones hVS-88 (C1) (infant) and hVS-228 (C2) (teenage) were obtained from anonymous control donors. iPSC reprogramming was done based on polycistronic construct with *OCT3/4*, *c-MYC*, *SOX2*, and *KLF4* [25]. The iPSC line (patient 2: line 417, MD5, P2) selected for this study was derived from a patient with mental retardation and seizures along with other TSC-related abnormalities. All cells were cultured in Essential 8 medium (Gibco) on Geltrex LDEV-free (Gibco). Generation of indirect contact iPSC–neuronal cultures for the two independent controls (1 batch of differentiations each) and one TSC2 patient line (2 batches of differentiations) was performed as described earlier [26] with slight modifications. Briefly, high-density hiPSC cultures were passaged onto Geltrex (GIBCO)-coated plates. When hiPSC cultures reached confluence, they were neural induced with Noggin (500 ng/ml; Peprotech), and SB431542 (10 μM; Stegment and Selleck chemicals). Neural rosettes were picked manually and cultured in neural maintenance medium (NMM) with FGF2 (20 ng/ml) and EGF (20 ng/ml; Peprotech) on PLO (20 μg/ml)/mouse laminin (20 μg/ml; both from Sigma) pre-coated plates. Neuronal cultures with a density of 62.5 K/2.0 cm^2^ were maintained in the medium contained neurobasal composition with brain-derived neurotrophic factor (BDNF) (20 ng/ml; Peprotech) and cAMP (1 μM; Sigma). At day 45 of differentiation, some cultures were treated with 10 nM rapamycin for the remainder of the cultures. To promote maturation, neurons were treated with CultureOne™ Supplement (Cat No. A3320201) at day 50 of differentiation for 2 days. At day 60 of differentiation, TSC2 patient and control neurons were plated on the multi-electrode arrays (MEAs). MEA analyses were performed on the combined results for the two controls and patient cultures since the variance within the control and the TSC2 groups were low.

### Multiple electrode arrays (MEAs)

All experiments were performed using CytoView MEA 24-well plates (M384-tMEA-24 W, 4X4 electrode grid). MEAs were first pre-treated with 0.1% polyethylenimine (Sigma) and incubated for 1 h at 37 °C. Day 60 neurons were plated as high density drop cultures (5000 cells/µl) containing 10 µg/ml laminin. After 1 h, conditioned medium was added into each MEA and after 24 h 0.5 ml of fresh BrainPhys media was added to each array. MEA cultures were maintained in 1:1 fresh to normal human astrocytes (NHA) condition BrainPhys media replaced every 3–4 days. Electrophysiological activity was recorded every 10 days using hardware (Maestro Pro complete with Maestro 768channel amplifier) and software (AxIS 1.5.2) from Axion Biosystems (Axion Biosystems Inc., Atlanta, GA). Channels were sampled simultaneously with a gain of 1000 × and a sampling rate of 12.5 kHz/channel. During the recording, the temperature was maintained constant at 37 °C. All active electrodes were included in the analysis. Before every recording, all recorded wells are rigorously quality controlled to check that the cells are not clumped or lifted off, and they also have to cover at least 80% of the electrodes. Any well that does not meet these criteria will be excluded from the analysis.

For drug treatment, LYN-1604 were purchased from Cambridge Bioscience (Cambridge, UK), rapamycin were purchased from Sigma-Aldrich (St. Louis, MO, USA), CNQX, APV, kainic acid and bicuculline from R&D System (R&D System Inc., Minneapolis, MN, USA), ACIAR and methyl-6,7-dimethoxy-4-ethyl-beta-carboline-3-carboxylate (DMCM) were purchased from Tocris (Bio-Techne Ltd.). Stock concentrations were diluted as required in 1 ml 1:1 BrainPhys:BrainPhys ACM. Medium containing diluted drugs were added to MEAs and subsequently incubated for 10 min at 37 °C in a standard 5% CO2 incubator environment unless otherwise stated. After recordings, medium was removed from MEAs and cultures were washed 3 times with PBS. 1 ml of fresh medium was then added, and cultures were incubated for 10 min as before. This medium was then removed and replaced with fresh medium and recorded again.

A Butterworth band-pass filter (with a high-pass cut-off of 200 Hz and low-pass cut-off of 3000 Hz) was applied along with a variable threshold spike detector set at 5.5 × standard deviation on each channel. Offline analysis was achieved with custom scripts written in MATLAB (available on request). Briefly, spikes were detected from filtered data using an automatic threshold-based method set at − 5.5 × σ, where σ is an estimate of the noise of each electrode based upon the median absolute deviation 1. Spike timestamps were analysed to provide statistics on the general excitability of cultures. Neuronal bursting was detected based on three parameters: inter-burst period longer than 200 ms, more than three spikes in each burst and a maximum inter-spike (intra-burst) interval of 300 ms. Network activity was illustrated by creating array–wide spike detection rate (ASDR) plots with a bin width of 200 ms. Heat map for spatially correlated neurons on the MEAs were generated with MATLAB imagesc function using in-house scripts. In the heat maps, a dark red–dark blue colour scale was used whereby shades of red and blue represented higher and lower correlations, respectively, for control or TSC2 neurons plated on the 16 electrode-grid.

### RNA extraction and quantitative (q)PCR

Cells were lysed with QIAzol Lysis Reagent (Life Technologies) and stored at -20 °C. Total RNA was extracted from hiPSCs-derived TSC and control neuron lysates using the miRNeasy mini kit (reference 217,004, Qiagen, Germany). For each sample, 1 µg of total RNA was reverse transcribed using the miScript II RT Kit (Qiagen). For qPCR analysis, QuantiTect SYBR Green PCR kit (Qiagen, Germany) was employed and samples were run on a StepOnePlus™ Real-Time PCR System (Applied Biosystems) following the manufacturer’s instructions. All reactions were performed in triplicate for each sample. The relative expression levels of the miRNAs and other genes were calculated using the 2^−ΔΔCT^ method [27], and the data were normalised to *GAPDH* and *Colrf43*. The primer sequences for all genes examined in the current study are listed in Additional file 1: Table S1.

### Statistical analysis

Prism 8.0 (GraphPad Software) was used for the statistical analysis. Data shown are the mean ± s.e.m. with *p* < 0.05 considered statistically significant. Two-tailed unpaired/paired *t* tests were used for comparisons between two groups. Data distribution was assumed to be normal, but this was not formally tested.

## Results

### *TSC2* patient iPSCs-derived neurons exhibit higher neuronal excitability but decreased synchronicity

In previous work, we reported enhanced neuronal excitability of iPSCs derived from ASD with *TSC1* or *TSC2* mutations [17]. In these experiments, mature neurons exhibited increased spontaneous calcium influx frequencies and an increased firing rate of TSC patient-derived neurons plated on MEAs as compared to control neurons [17]. In the current study, we have investigated the synchronisation and connectivity of neuronal network activity of two independent control lines and one patient that was lacking functional *TSC2*, possessing a single nucleotide duplication (1563dupA) leading to a frameshift mutation H522T [17]. Several iPSC clones were obtained from each patient and control fibroblast line and verified by sequencing and characterised by immunostaining as previously shown [17].

**Fig. 1:**
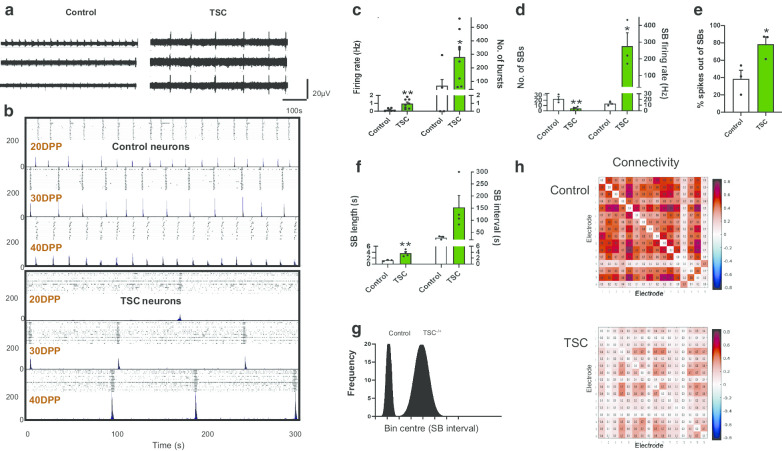
Changes in neuronal activity of patient and control neurons recorded with multi-electrode arrays (MEAs). **a** Representative voltage traces from three electrodes of the same MEA culture for control and *TSC2* neurons at 40DPP (DPP = days post plating). Traces from *TSC2* neurons show high levels of activity compared to control neurons, as expected from previous observations [17]. **b** Development of synchronised bursting across the array at 20, 30 and 40DPP for control (top) and *TSC2* neurons (bottom). For each time point, upper panel shows a raster plot and lower panel shows an array-wide synchronised detection rate (ASDR) plot. Vertical-scale bars = 200 spikes per 200 ms bin, following 5 min of recording. **c** Basal excitability of control and *TSC2* neurons showing average spike firing rate and number of single unit bursts detected. Changes in **d** synchronised burst (SB) activity and the number of spikes in individual SBs, **e** the spikes outside of SBs and **f** SB length and interval in control and *TSC2* models. **g** Frequency distribution analysis represents the variation in the SB intervals of control neurons *TSC2* neurons. **h** Connectivity correlation matrices heat map for the control and TSC2 neurons on the MEAs, and colours represent the correlation in the firing rates across the indicated electrode. Correlation matrices are calculated for 16 electrodes in control and *TSC2* neurons plated on the MEAs. Values greater than zero represent positive correlation, while values below zero represent negative correlation. All plots show means ± SEM. *p < 0.05, **p < 0.01 following unpaired t-tests. Number of recorded wells = 6 for the control and 8 for TSC2 culture

Control and patient iPSCs were differentiated into functional neurons, and at ~ day 60 of differentiation neurons were plated on the MEA plate to record their activities. Spike detection from filtered raw voltage recording was generated from each electrode via the threshold-based method of QuianQuiroga and colleagues [28], and following quality control spikes were time-stamped for subsequent analysis. As previously reported, spontaneous activity of cultures on MEAs differed between control and *TSC2* mutated patient neurons (Fig. 1a, b) with increased spontaneous firing rates and higher total numbers of single unit bursts in *TSC2* neurons, with 0.9 ± 0.2 Hz, 273.1 ± 68.81 bursts, respectively, compared to the control values of 0.16 Hz ± 0.07 Hz, 57.67 ± 46.76 bursts, respectively (*p* < 0.01, *p* < 0.05, unpaired *t* test, Fig. 1c).

After 20 days on MEAs, synchronised bursting emerges, where multiple electrodes across the array simultaneously detect burst firing. Control neuronal cultures develop a regular pattern of synchronised bursts (SBs) separated by intervals of similar length, consistent with that previously reported [29] (Fig. 1a, b, Additional file 1: Fig. S1). In *TSC2* mutated patient neurons, there was a significant reduction in SB frequency (Fig. 1a, b). For example, at 40DPP (days post plating) the number of SBs was significantly lower in *TSC2* neurons, 4.5 ± 1.55 SBs as compared to control neurons, 22.67 ± 4.4 SBs (*p* < 0.01, unpaired *t* test, Fig. 1d). Consistent with elevated general spontaneous activity in *TSC2* neurons, the firing rate within the *TSC2* SBs was higher; for example, at 40DPP the SB firing rate in *TSC2* neurons was 282.3 ± 77.04 Hz as compared to 13.67 ± 2.33 Hz in control neurons (*p* < 0.05, unpaired *t* test, Fig. 1d). This suggests that although a higher intrinsic neuronal activity is present in *TSC2* neurons, it is not reflected in increased synchronised activity within the neuronal network, and in fact there exists a previously undetected deficit in network behaviour of the patient neurons.

To probe further, we investigated how the pattern of neuronal firing differed between control and *TSC2* neurons. As would be predicted, the percentage of firing spikes occurring outside SBs was significantly higher in *TSC2* neurons (78.44 ± 8.5% in *TSC2* neurons as compared to 38.5 ± 9.8% in control neurons, *p* < 0.05, unpaired *t* test, Fig. 1e). However, when SBs did occur in the *TSC2* neurons, they persisted for a longer period compared to those in control neurons; approx. 2.9-fold longer at 40 DPP than those in the control (1.25 ± 0.13 s, 3.65 ± 0.54 s (*p* < 0.01, unpaired *t* test Fig. 1f). This is accompanied by a substantial increase, approx. 5.8-fold, in the time interval between SB for *TSC2* neurons compared to controls (151.5 ± 50.21 v 26.67 ± 4.37 s at 40DPP, *p* = 0.09, unpaired *t* test), (Fig. 1f). Due to higher variation in SB interval observed for*TSC2* neurons, the increased SB interval did not reach a *p* < 0.05 threshold, conventionally considered as statistically significant. To assess the increased variation with the *TSC2* dataset in comparison with the control, we plotted the range of SB interval lengths for both control and TSC2 neurons (Fig. 1g). SBs in control cells exhibited a regular defined pattern with intervals that are tightly clustered around the mean interval length, in contrast the distribution of the interval times in *TSC2* neurons were widely dispersed with a range approx. fivefold greater than control (Fig. 1g). Combining these data indicate that although the SBs of *TSC2* neurons have more persistent and higher firing rates, their spontaneous frequency is significantly reduced and a disorganised. This pattern is well illustrated in Fig. 1b.

This loss of synchronicity seen in the *TSC2* neuronal networks is suggestive of a reduced connectivity between groups of neurons. To pursue this observation, we interrogated how the neuronal spatial connectivity may differ between the control and *TSC2* neurons plated on MEAs by plotting correlation matrices between all electrodes in the MEA. In control neurons, we observed a high firing correlation between the majority of the electrodes in the cultures, represented as red and dark red pixels in Fig. 1h, indicative of high level of neuronal connectivity. The firing correlation is substantially reduced for *TSC2* plated neurons, showing a loss of neuronal connectivity.

### Pharmacological profiling of *TSC2* patient-derived neuronal networks

**Fig. 2:**
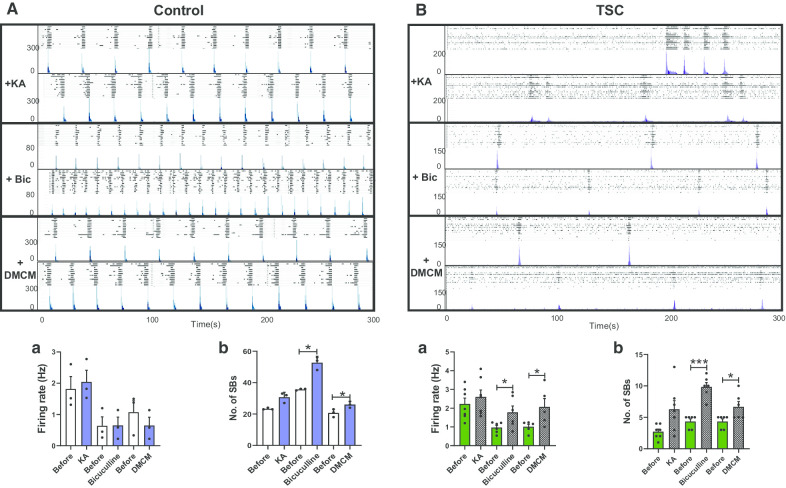
Pharmacological profiling of the network activity of *TSC2* neurons. Typical synchronised burst patterns shown by raster plot (upper panel) and ASDR plots (lower patterns) for Control (**A**) and TSC2 (**B**) neurons, treated with 1 μM kainic acid (KA), 10 μM bicuculline (Bic) or 1 μM methyl-6,7-dimethoxy-4-ethyl-beta-carboline-3-carboxylate (DMCM). Plots (underneath) show mean ± SEM of **a** spike firing rate (Hz) and **b** number of synchronised bursts (SB) for all drugs. *p < 0.05, ***p < 0.001 following paired t-tests. Number of recorded wells = 3–10

To establish whether the SB firing patterns observed in *TSC2* neurons arise due to changes in synaptic activity as previously reported in our control neurons [29], we probed our cultures with agents that modulate glutamate or GABA signalling. The agents were applied after 50DPP when SBs patterns had fully established. In agreement with what we found previously in control neurons inhibition of glutamate signalling via an AMPA receptor antagonist (CNQX) or an NMDA receptor antagonist (APV) lead to a complete abolition of the SB in *TSC2* cultures (Additional file 1: Fig. S2). Previously, it has been reported that the glutamate mimic, kainic acid (KA) increased the number of SBs [30], and we found that any increase in control or *TSC2* neurons did not reach statistical significance (Fig. 2A, B). Probing *TSC2* or control cultures with bicuculline or DMCM (6,7-dimethoxy-4-ethyl-beta-carboline-3-carboxylate methyl ester), drugs that antagonise GABA receptors [31] increased the number of SBs (Fig. 2A, B). As in all of the previous cases, the patterns of the SBs rapidly recovered after washing out the drug (data not shown). Taken together, these findings indicate that consistent with observations in control iPSCs-derived neurons, the SB patterns detected in *TSC2* neurons arise via synaptic activity and that AMPA, NMDA and GABA signallings are all required for their neuronal network activity.

### *TSC2* patient iPSC-derived neurons show an excitatory/inhibitory synaptic marker imbalance

**Fig. 3:**
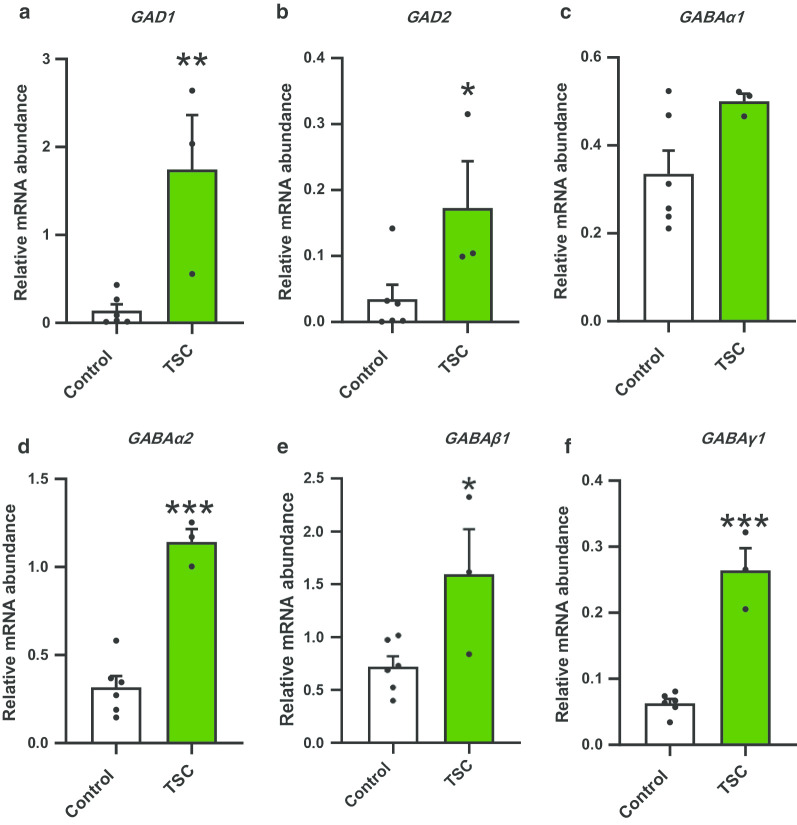
Expression analysis of genes encoding GABA signalling components. Analysis of a panel of inhibitory GABA signalling genes (**a**–**f**: GAD1, GAD2, GABAa1, GABAa2, GABAb1, GABAg1) in control and TSC2 neurons at 60DPP. Data are represented as means ± SEM. *p < 0.05, **p < 0.01, ***p < 0.001 following unpaired t-tests, N = 6 for control and 3 for TSC neurons. Also see Table 1

As the SB firing patterns in *TSC2* neurons are dependent on synaptic activity, we interrogated whether the abnormal network phenotype observed in *TSC2* neurons correlated with a potential excitatory/inhibitory imbalance. We used transcription analysis to probe possible modes of change in (1) excitatory–inhibitory cell ratio, (2) synaptic gene expression and (3) mediators of glutamate-GABA signalling. At the end of the MEA recording period ~ 60DPP, mRNA levels in *TSC2* and control neuron cultures were quantified by qRT-PCR (Table 1, Fig. 3, Additional file 1: Fig S3).

**Table 1:** Gene expression changes between control and TSC patient neurons

Target gene	RNA abundance (mean ± SEM)		Change
	Control neurons	TSC neurons	
**GABAergic cells**			
*DLX1*	0.016 ± 0.009	0.002 ± 0.0004	
*DLX2*	0.03 ± 0.01	0.05 ± 0.01	
*LHX6*	0.08 ± 0.02	0.11 ± 0.02	
*VGAT*	0.03 ± 0.01	0.04 ± 0.01	
**Synaptic**			
PSD95	0.21 ± 0.05	0.12 ± 0.02	
Synaptophysin	0.07 ± 0.01	0.11 ± 0.03	
Homer1	0.37 ± 0.07	0.43 ± 0.05	
**GABA/glutamate signalling**			
*GAD65*	0.034 ± 0.02	0.17 ± 0.07*	↑
*GAD67*	0.13 ± 0.07	1.74 ± 0.61**	↑
*GABAα1*	0.33 ± 0.05	0.50 ± 0.02	
*GABAα2*	0.31 ± 0.06	1.14 ± 0.07***	↑
*GABAβ1*	0.72 ± 0.09	1.59 ± 0.43*	↑
*GABAγ1*	0.062 ± 0.006	0.26 ± 0.03***	↑
*Grin2a*	0.27 ± 0.08	0.14 ± 0.007	
*Grin2b*	1.35 ± 0.26	0.82 ± 0.06	
*Grin3A*	0.13 ± 0.036	0.29 ± 0.05*	↑
*GRIN1*	0.07 ± 0.007	0.18 ± 0.04**	↑
*GRIA1*	1.03 ± 0.14	3.51 ± 1.04*	↑
*Vglut1*	0.06 ± 0.009	0.004 ± 0.002**	↓
Vglut2	0.1 ± 0.04	3.11 ± 0.85**	↑

*p <  0.05; **p < 0.01; ***p < 0.001 (unpaired *t* test)

Expression of cell and regional-specific markers for GABAergic cells, *DLX1*, *DLX2*, *LHX6* and *VGAT* was used to probe the proportion of inhibitory neuron cells. In *TSC2* cultures, elevated GABA signalling would be expected to suppress SB formation and frequency. There was no significant expression change of these cell markers between *TSC* and control neurons (Table 1, Additional file 1: Fig. S3), suggesting that major changes in cell-type proportions are unlikely. Previously, we observed no significant differences in neuronal morphology or synaptic number in *TSC2* patient neurons [17], and consistent with these earlier studies there was no significant differences in expression of the postsynaptic density protein genes *PSD95* and *Homer1* or the presynaptic marker *synaptophysin* [35] (Table 1, Additional file 1: Fig. S3).

In contrast, significant differences were observed in expression of genes encoding enzymes and receptor proteins associated with glutamate-GABA signalling. *GAD1* and *GAD2*, which encode the glutamic acid decarboxylases GAD67 and GAD65 [32], showed a statistically significant increase of approximately 5- and 20-fold changes, respectively, in *TSC2* neurons as compared to the controls (Table 1, Fig. 3a, b), (*p* < 0.05, *p* < 0.01, unpaired *t* test). Likewise, expression of the postsynaptic *GABA*_*A*_ receptor subunits *α1*, β2 and γ1 (GABAα2, GABAβ1 and GABAγ1) were also elevated by six, four and four-fold, respectively, in *TSC2* neurons (Table 1, Fig. 3d–f, *p* < 0.05,* p* < 0.001, unpaired *t* test). In addition, expression of the glutamate receptor genes *GRIN1, GRIN3A, and GRIA1* [33, 34] and the presynaptic vesicular glutamate transporter *VGLUT2,* also showed a clear increase (Table 1, Additional file 1: Fig. S3), (*p* < 0.05, *p* < 0.01, unpaired *t* test). *GRIN2A* and *GRIN2B* genes had no significant change, and *VGLUT1* was very strongly decreased, although its mRNA abundance is relatively low (~ 0.05) even in control neurons. These results are indicative of disruption of both GABA and glutamate signalling. Elevation of glutamate signalling could explain the neuronal hyperexcitability of *TSC*2 model, while increased GABA signalling would be expected to suppress synchronised neuronal network behaviour [29].

### Chronic inhibition of mTORC1 pathway has no effect on the neuronal network behaviour in *TSC2* patient neurons

**Fig. 4:**
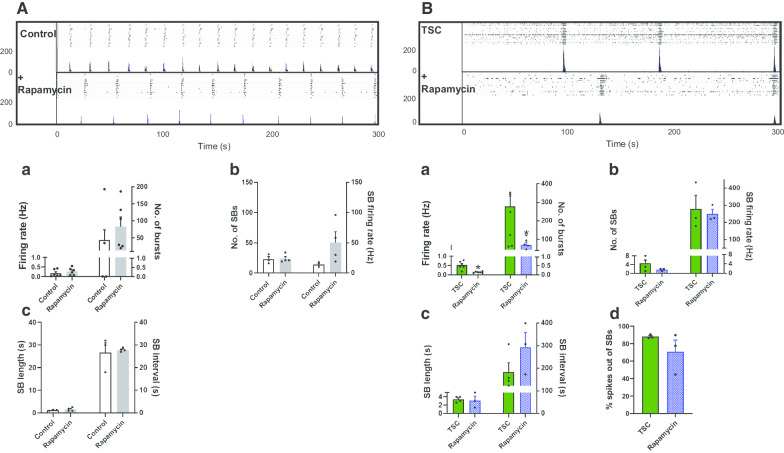
Effects of chronic treatment with rapamycin on *TSC2* neuronal activity. Raster (upper panels) and ASDR plots (lower panels) of a single neuronal MEA culture at 60 DPP in the absence (**A**) and presence of rapamycin (10 nM) (**B**). Vertical-scale bar = 200 spikes per 200 ms bin. Underneath show changes in **a** basal excitability, **b** synchronised burst (SB) activity and the number of spikes in individual SB, **c** SB length and interval and **d** spikes firing outside of a SB. All plots show means ± SEM. *p < 0.05 following unpaired *t* tests. Number of recorded wells = 6

As loss of *TSC2* increases mTORC1 activity, we interrogated whether longer-term inhibition of mTORC1 by rapamycin may rescue the neuronal network defects phenotype detected in *TSC2* neurons. Control neurons and *TSC2* neurons were treated with 10 nM rapamycin from day 45 of differentiation, and neuronal activity of control and *TSC2* mutant cells was examined at day 60 on MEAs (Fig. 4A, B). As seen previously, there was a significant decrease of neuronal hyperactivity in the basal neuronal activity (spike firing rate and total number of bursts) in *TSC2*-derived neuronal cultures, reaching a comparable level seen in the control firing rate, i.e. a decrease from 0.52 ± 0.09 Hz to 0.13 ± 0.02 in the presence of rapamycin (*p* < 0.05, unpaired *t* test). However, there was no significant alteration in the overall neuronal activity or synchronicity in *TSC2* patient-derived neurons, or even those of the control, with rapamycin (Fig. 4B). Given that chronic inhibition of the mTORC1 pathway did not increase the number of SBs, we tested whether short-term treatment with rapamycin may have an effect. *TSC2* neurons were treated with rapamycin for 24 h and then the neuronal network activity was recorded. Similar to that seen in *TSC2* neurons chronically treated with rapamycin, short-term rapamycin treatment decreased the basal excitability of *TSC2* neurons, but again it had no effect on the SB number, SB length or percentage of spikes outside SB, although we did observe a small change in the mean interval between SB (Additional file 1: Fig. S4). In conclusion, neither long- nor short-term rapamycin treatment had a major effect on the abnormal network pattern seen for *TSC2* neurons.

### Suppression of mTORC1 via ULK1 enhances the neuronal synchronicity in *TSC2* patient-derived neuronal networks

**Fig. 5:**
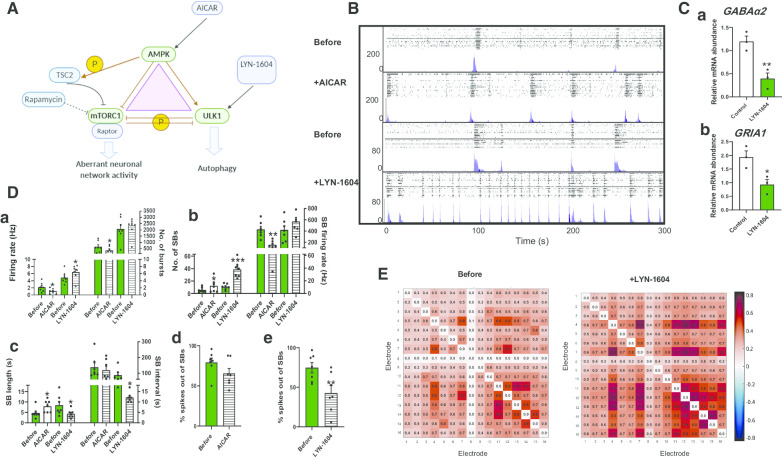
Effects of AICAR and LYN-1604 on *TSC2* neuronal activity. **A** Schematic to show the relationship of TORC1 to AMPK and ULK1. **B** Raster (upper panel) and ASDR (lower panel) plots of recordings of the same MEA showing 24-h exposure to AICAR (1 mM) and LYN-1604 (2 μM). **C** Gene expression analysis of the genes encoding GABAa2 (**a**) and GRIA1 (**b**) in control cells following 24-h exposure to LYN-1604 (2 μM). **D** Network activity following 24-h drug treatment of *TSC2* neurons in the absence and presence of AICAR and LYN-1604 in the absence and presence of AICAR and LYN-1604 showing **a** basal excitability, **b** synchronised burst (SB) activity and the number of spikes in individual SBs, **c** SB length and interval and **d, e** the spikes out of SBs, presented as percentage of the response before the drugs treatment. **E** Correlation matrices heat map for TSC2 neurons in the absence and presence of LYN-1604, colours represent the correlation in the firing rates across the indicated electrode. Correlation matrices are calculated for 16 electrodes in control and *TSC2* neurons plated on the MEAs. Values greater than zero represent positive correlation, while values below zero represent negative correlation. All plots show means ± SEM. *p < 0.05, **p < 0.01 following paired t-tests. Number of recorded wells = 7

In many cell types, rapamycin is reported as being a poor inhibitor of mTORC1 due to incomplete allosteric inhibition [36]. However, TORC1 activity is also inhibited by the action of the two kinases AMPK and ULK1 (Fig. 5A). *TSC2* cultures were probed with AICAR, an AMPK activator, for 24 h and found to decrease*TSC2* neuronal excitability (Fig. 5B, D). Unlike rapamycin, AICAR significantly increased the number of SBs and SB length and decreased the SB firing rate in *TSC2* neurons (Fig. 5Db, Dc). However, it did not alter the SB interval (Fig. 5Dc) or decrease the number of firing spikes outside the SBs (Fig. 5Dd). In control neurons, AICAR had no effect on the neurons basal activity and in fact reduced the number of SB following 24 h of treatment (Additional file 1: Fig. S5).

To seek a more effective reversal of the aberrant phenotype of *TSC2* neurons, we investigated LYN-1604, a small molecule which is a potent and selective ULK1 activator [37, 38] and should inhibit mTORC1 through direct phosphorylation of Raptor [23, 39]. A preliminary screen of LYN-1604 showed that in control neurons, it reduced expression of *GABAα2* and *GRIA1* with 24 h of treatment (Fig. 5C), an indication that it may target both GABA and glutamate signalling in the *TSC2* patient-derived neurons. Consistent with this possibility, MEA cultures of *TSC2* patient-derived neurons treated with LYN-1604 for 24 h showed a significant increase in the number of SB, comparable to that observed in control neurons (Fig. 5Db). It also reduced the SB length and interval and significantly decreased the number of firing spikes outside the SBs (Fig. 5Dc, De), whereas the effect of LYN-1604 on the number of SBs in the control neurons is yet to be determined.

As AICAR and LYN-1604 both showed degrees of improvement in synchronicity in *TSC2* neurons, we examined whether probing the culture with any of these drugs would alter the neuronal spatial connectivity. We analysed the correlation matrices between all electrodes of the MEAs for *TSC2* neurons before and after treatment with AICAR and LYN-1604. While treatment with AICAR had no detectable effect on connectivity matrices of the *TSC2* neurons plated on MEAs (Additional file 1: Fig. S5c), treatment with LYN-1604 improved the correlation between several electrodes. This effect was presented by increasing the red and dark red pixels between several electrodes in the presence of LYN-1604, an indicative of increasing the neuronal connectivity (Fig. 5E). Taken together, these results show that the defective *TSC2* neuronal networks may be partially restored by induction of AMPK but are substantially restored by ULK1 activation by LYN-1604.

## Discussion

In this report, we examine the abnormal neuronal network behaviour observed in *TSC2* patient iPSC-derived neurons harbouring a *TSC2* mutation. We find that *TSC2* patient neurons exhibit hyperexcitability but a lower degree of synchronicity. We illustrate for the first time an approach to reverse the defects in TSC2 neuronal synchronicity through the activation of AMPK and ULK1, which would activate autophagy and mechanistically inhibit mTORC1 through phosphorylation of Raptor. The aberrant network behaviour of *TSC2* patient-derived neurons could be explained by an excitatory/inhibitory synaptic imbalance. The higher level of genes for GABA signalling at the synapse may shift the excitatory–inhibitory balance towards inhibition and could account for the reduction in the SB frequency, the disorganisation in SB patterns and the reduction in neuronal spatial connectivity.

In mouse models, loss of *Tsc1* from all neurons in local cortical circuits or the hippocampus led to neuronal hyperexcitability measured by increased spontaneous neuronal activity and induction of a seizure-like phenotype [40, 41]. The latter phenotype was shown to be due to an imbalance in excitation and inhibition and a reduction in inhibition onto *Tsc1* KO pyramidal neurons [41]. Knockout of *Tsc1* in mouse neurons also showed clinical and electrographic seizures both spontaneously and under physical stimulation, and they also exhibited enlarged cortical and hippocampus neurons [42]. *TSC*1 KO mice presented with short spike bursts, spontaneous periods of desynchronisation or frequent high-amplitude sharp waves [42]. We have previously examined the effect of losing one copy of *TSC1* or 2 on neuronal morphology and excitability. We showed that *TSC2*-derived neurons have common neuronal defects caused by both autosomal dominant *TSC1* and *TSC2* mutations. We found that neuronal hyperexcitability of *TSC* patient-derived cells was reversed by rapamycin [17]. Subsequently, Winden et al. studied the abnormalities in the neuronal excitability resulted from *TSC2* mutation. They used induced expression NGN2 to generate excitatory neurons from *TSC2*^−/+^, *TSC2*^−/−^ and *TSC2*^+/+^ cells [16]. While their results replicate those previously observed for *TSC* neuronal hyperexcitability, they did not examine the network behaviour of *TSC2* mutants. In particular, Winden et al. examined only excitatory neurons generated by their induced development protocol. Here, our protocol generates a mix of excitatory and inhibitory expressing GAD65/67 and is more representative of human cortical development [29, 43].

Our current data are consistent with our previous findings and other reports of hyperexcitability of neurons with *TSC2* loss of function mutations [16, 17], but also show a reduction of network synchrony and connectivity. Previously, Sundberg et al. found that *TSC2* patient neurons exhibited reduced synaptic activity, measured by a decrease in the number of functional glutamatergic synapses in relation to controls with no effect on the glutamate receptor properties [11]. In our work, the reduced synchronicity could be due to increased GABAergic signalling in this model. The regulation of coordinated network firing by GABAergic interneurons is well documented, and increased GABA activity could prevent the coordination between active and non-active periods in the culture, leading to a significant elevation in the number of spikes outside the SBs. This leads to network disorganisation, manifest as a high number of random and uncorrelated spikes. The large reduction in the number of SBs detected in the *TSC2* model was associated with an increase in the burst length, a feature consistent with previous reports in both animal and human models [16, 44].

Our pharmacological profiling demonstrated that consistent with that already shown in control human iPSC-derived neurons and in rodent neurons, glutamate and GABA signalling are the primary drivers for the network activity in *TSC2* neurons [29]. The SBs detected in *TSC2* neurons were completely abolished when NMDA or AMPA receptors were inhibited. Conversely, inhibition of GABA_A_ receptors increased the number of SBs and decreased the SB intervals. This indicates that, as for non-patient cells, in our *TSC2* patient cells glutamate signalling is the driver of neuronal network activity while GABA signalling shapes it. Importantly, shifts in excitation or inhibition signalling cause an alteration in the excitability of local circuits lead to an impairment of information processing in the brain, which is a potential pathophysiological mechanism in several neuropsychiatric disorders including ASD, schizophrenia and epilepsies [45]. Murine *Tsc1* KO hippocampal hyperexcitability was shown to result from an increase in the balance of inhibitory versus excitatory synapses [41].

The hypothesis that the aberrant network behaviour observed in *TSC2* neurons could be because an increase in inhibitory/excitatory synaptic activity is supported by RNA analysis of a panel of excitatory and inhibitory synaptic markers. While the genes encoding the markers for GABAergic cell types and synaptic components including *synaptophysin* and *PSD95*, did not differ from those of the control neurons [46, 47], the expression levels of presynaptic inhibitory markers and the postsynaptic *GABA*_*A*_ receptor subunits *α2, β1* and *γ1* were significantly elevated in our *TSC2* model. Likewise, expression of the glutamatergic markers, *GRIN1, GRIA1 GRIN3A* and *VGLUT2* was increased, suggesting that the aberrant network behaviour detected in this model could be attributed to the excitatory/inhibitory synaptic imbalance. In line with this observation, hiPSC-derived neurons treated with GABA exhibited a significant reduction in the neuronal synchronicity [29]. Additionally, one potential mechanism of reduced synaptic activity in *TSC* patient-derived neurons is alteration in the level of FMRP targets [48]. In here we found that the expression of FMRP targets, *GABA*_*A*_ receptors and *GAD1* and *GAD2* were significantly elevated [48]. Suggesting that reduced synchrony in *TSC2* patient-derived neurons could be due to dysregulation of FMRP targets which can be altered due to *TSC2* mutation.

Interestingly, the number of SBs in *TSC2* neurons did not increase following rapamycin treatment, indicating that rapamycin was not able to improve the *TSC2* neuronal network activity. Previous studies showed that while the involvement of rapamycin in *TSC2* patients control tumour growth, its efficacy in managing neuropsychiatric-associated behaviour in *TSC2* patients has remained unclear [49]. Rapamycin failed to reverse the enhanced proliferation and altered neurite outgrowth; phenotypes inherent to *TSC* KO NPCs [50]. As rapamycin only particularly rescues the *TSC2* neuronal phenotype, having little effect on the network deficit, we targeted aberrant synchronicity and connectivity deficits with alternative approaches via upstream *TSC* activation or by manipulating the mTORC1 at the molecular level. We found that phosphorylation of the remaining copy of *TSC2* in our model via AMPK activation increased the number of SBs. However, the number of spontaneous spikes outside of the SBs was not altered, suggesting that the culture was still disorganised and less connected. The ULK1 activator, LYN-1604, inhibits mTORC1 signalling through direct phosphorylation of Raptor [19]. We observed that ULK1 activation with LYN-1604 not only caused an increase in the number of SBs to a level comparable to that seen in control neurons, it also significantly decreased the number of spikes outside the SBs, suggesting a more connected culture. However, LYN-1604 treatment may need further refinement as the SB length in the presence of LYN-1604 was ~ 4 time longer than those observed in control cells.

High mTORC1 activity prevents ULK1 activation by phosphorylating ULK1 at Ser-757 and disrupts its interaction with AMPK, the TSC upstream activator [19]. ULK1 and autophagy is compromised by mTORC1 hyperactivity. However, treatment with LYN1604 would restore these defects through the reactivation of autophagy that would help restore homeostatic balance to these *TSC2* cells (Fig. 5A). Interestingly, rapamycin would be more effective at activating ULK1 at a higher dose of ~ 50 nM but not at the dose we used in the current study (at 10 nM), which is believed to be selective at mTORC1, iC50 = 20 nM and carried no effect on the patterns of our control neuron firings (Fig. 4A) [51]. Furthermore, rapamycin is an allosteric inhibitor of mTORC1 that cannot completely inhibit all the mTORC1-mediated phosphorylation events in cells, as is apparent by rapamycin resistant phosphorylation sites within 4E-BP1 [36]. Autophagy induction is also resistant to rapamycin treatment in many cell types, so it is very likely that the 10 nM rapamycin treatment in this study was insufficient to induce autophagy.

## Limitations

A limitation in our study is the number of *TSC2* patients examined. Although a robust and consistent phenotype is observed across multiple independent iPSC cultures on different plates, the results are based on one patient. There may be more subtle differences between patients with different *TSC2* mutations or differences of polygenic background within their genomes. This may affect the severity of the network deficit or the pharmacological response between *TSC2* patients.

## Conclusions

Taken together, we have used pharmacological approaches to rescue the aberrant neuronal network phonotype linked to TSC2 hypofunction. We have shown that disruption of mTORC1 signalling via ULK1 activation as well as phosphorylation of the remaining copy of TSC2 via AMPK is able to ameliorate synaptic dysfunction in *TSC2* patient-derived neurons. This approach can be used for future development of personalised therapies for these patients.

## Supplementary information

**Additional file 1: Table S1, Fig S1 and S2**. Pharmacological profiling of neuronal networks derived from ASD patient iPSC with a TSC2 mutation.

## Data Availability

The datasets used and/or analysed during the current study are available from the corresponding author on reasonable request.

## Abbreviations

AMPK: AMP-dependent protein kinase 1
ASD: Autism spectrum disorder
ASDR: Array–wide spike detection rate
hiPSCs: Human-induced pluripotent stem cells
MEA: Multi-electrode array
mTORC1: Mechanistic target of rapamycin complex 1
qPCR: Quantitative polymerase chain reaction
SB: Synchronised burst
TSC: Tuberous sclerosis complex
ULK1: Unc-51 like Autophagy Activating Kinase 1
