# Supplementary material for: Pharmacological intervention to restore connectivity deficits of neuronal networks derived from ASD patient iPSC with a TSC2 mutation
Source: Mol Autism. 2020 Oct 19;11:80. doi: 10.1186/s13229-020-00391-w (PMC7574213; doi:10.1186/s13229-020-00391-w)
Supplement: Supplementary file 1 — Additional file 1: Table S1, Fig S1 and S2. Pharmacological profiling of neuronal networks derived from ASD patient iPSC with a TSC2 mutation. [file 13229_2020_391_MOESM1_ESM.pdf]

Pharmacological intervention to restore connectivity deficits of neuronal networks derived from ASD patient iPSC with a TSC2 mutation

Mouhamed Alsaqati, Vivi M Heine, and Adrian J Harwood

Supplementary Information

Table S1

Primers for gene quantification.

Gene name	Forward (5'-3') &Reverse (5'-3')
GAD1	GCCAGACAAGCAGTATGATGT & CCAGTTCCAGGCATTTGTTGAT
GAD2	GGCTTTTGGTCTTTCGGGTC & GCACAGTTTGTTTCCGATGCC
$GABA_A α1$	GGATTGGGAGAGCGTGTAACC & TGA AACGGGTCCGAAACT G
$GABA_A α2$	GTTCAAGCTGAATGCCCA AT & ACCTAGAGCCATCAGGAGCA
GRIN2a	TGGACGTGAACGTGGTAGC & CCCCCATGAATGCCCAAGAT
GRIN2b	TTCCGTAATGCTCAACATCATGG & TGCTGCGGATCTTGTTTACAAA
GRIN3a	ACACGGCAAACTTGGCTGCTGT & CTTCAGCACTGCTTTCTCGGAC
GRIA1	GGTCTGCCCTGAGAAATCCAG & CTCGCCCTTGTCGTACCAC
GRIN1	CGCCGCTAACCATAAACAAC & GGGGAATCTCCTTCTTGACC
VGLUT1	CGACGACAGCCTTTTGTGGT & GCCGTAGACGTAGAAAACAGAG
PSD95	GATGTGTGGGTTGTCAGTGC & AGCCCCAGGATATGAGTTGC
Homer1	GCCAAGGGCTGAACCAACT & TGTTTCACTTGGGAGTGG
Synaptophysin	TGGTGTTCGGCTTCCTGAA & GCGGCCCAGCCTGTCT
GAPDH	AGGCTGGGGCTCATTTG & CAGTTGGTGGTGCAGGAG
Colrf43	GGCATCCCCGTTCCTTAATGG & TACACCCTTGAAAGGCGTACT
VGLUT2	GAGAGGAGTAGACTGGCAACCA & CTGAAGACCAGCCAGTGTACTG
VGAT Q	GGACTCGTACGTGGCCATAG & AGCTCGATGATCTGCGCTAC
LHX6	ACAGATCTACGCCAGCGACT & CATGGTGTCGTAGTGGATGC
DLX1	CATCAGTTCGGTGCAGTCCTAC & CCTTGCCATTGAAGCGCACTTC
DLX2	ACTACCCCTGGTACCACCAGAC & TCTGCTCTCAGTCTCTGGCGAGT TCTC

Fig S1

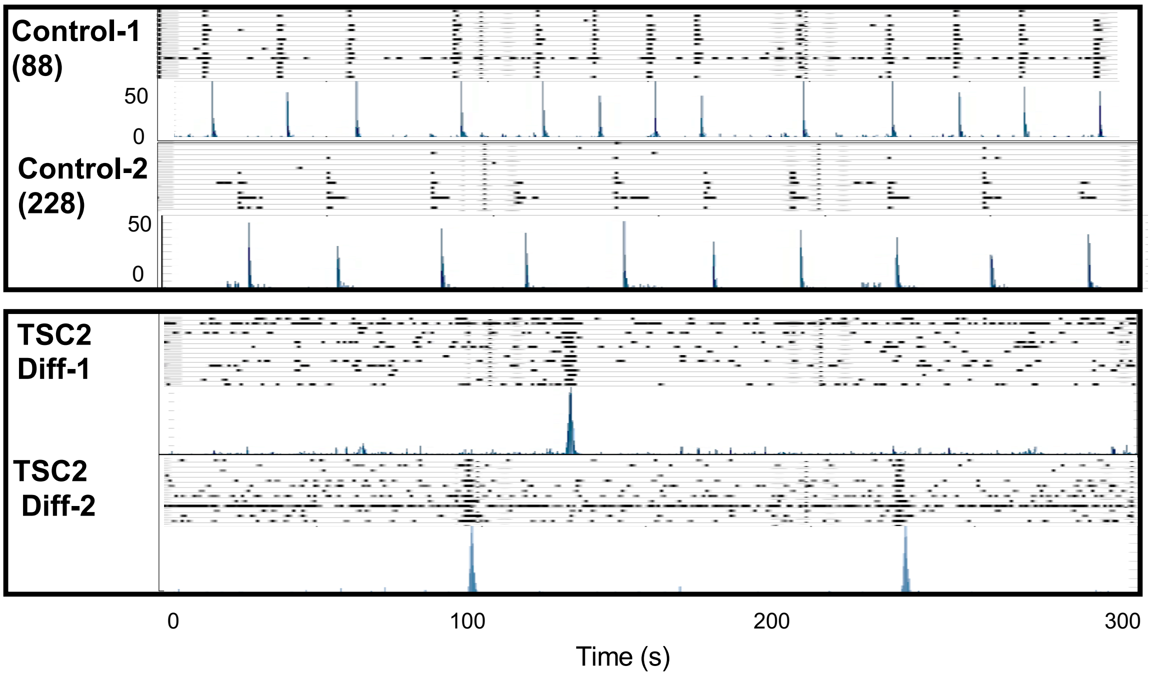

Reproducibility of synchronised bursting across at 40DPP for control (top) and *TSC2* neurons (bottom). For each time point, upper panel shows a raster plot and lower panel shows an Array-wide Synchronised Detection Rate (ASDR) plot. Vertical scale bars = 50 spikes per 200 ms bin for control and 80 spikes per 200 ms bin for *TSC2* neurons, following 5min of recording.

Fig S2

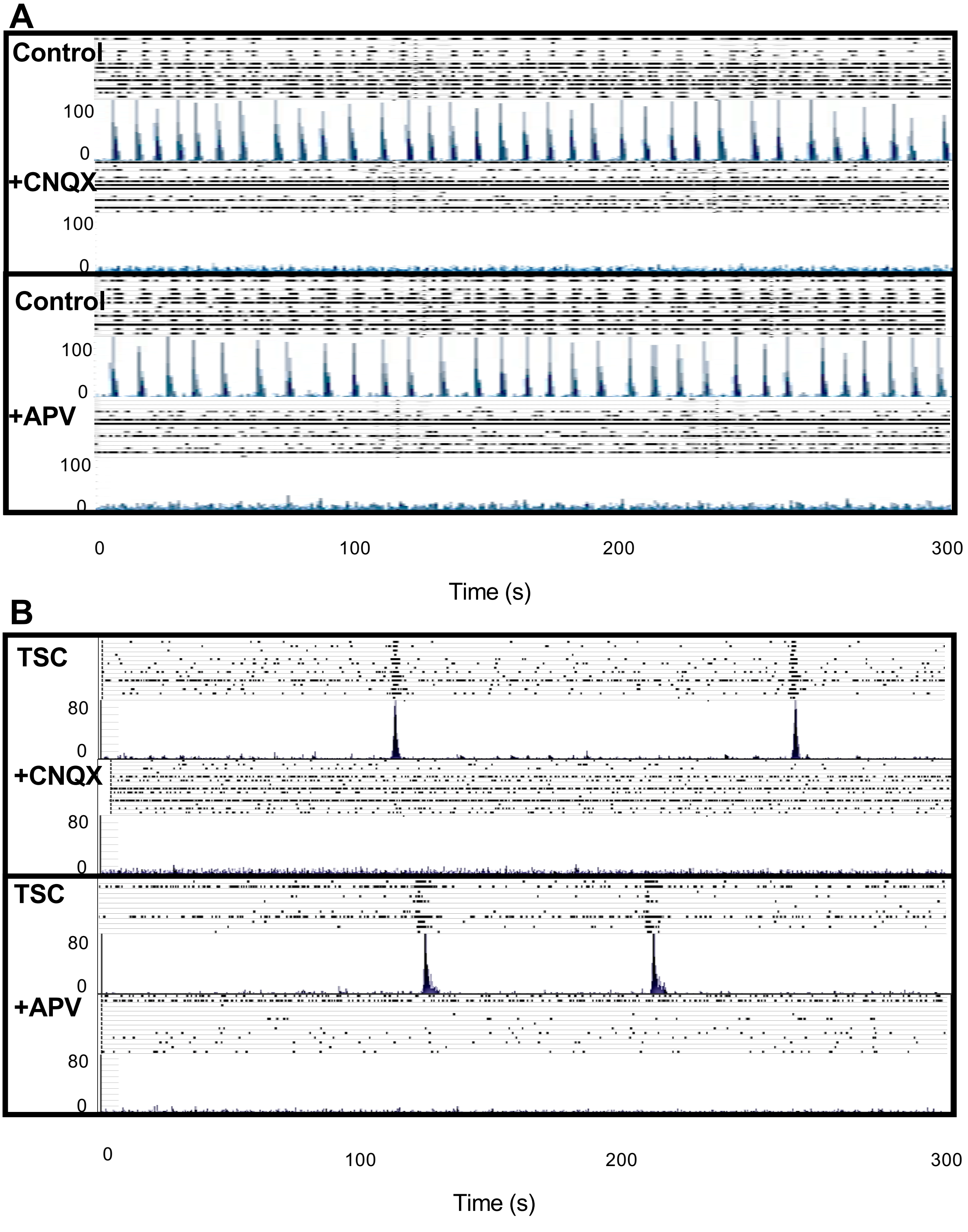

Pharmacological profiling of the network activity of Control and *TSC2* neurons. Raster (upper panel) and ASDR (lower panel) plots of recordings of the same MEA showing the culture-wide response to acute exposure of CNQX (50 µM) and APV (50 µM), Vertical scale bar = 100 spikes per 200 ms bin for control and 80 spikes per 200 ms bin for *TSC2* neurons, following 5min of recording.

Fig S3

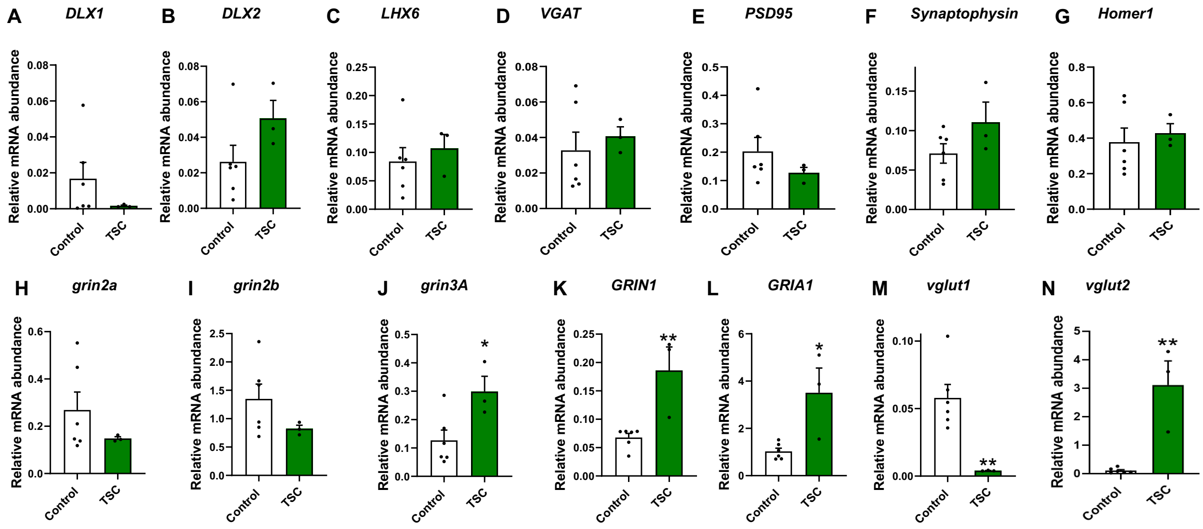

Expression Analysis of genes encoding regional specific markers for GABAergic cells (A-D: *DLX1*, *DLX2*, *LHX6* and *VGAT*), synaptic proteins (E-G: *PSD95, Homer1* and *synaptophysin*), and glutamate signalling components (H-N: *GRIN2A, GRIN2B,GRIN3A, GRIN1, GRIA1, VGLUT1 and VGLUT2*) in control and *TSC2* neurons at 60DPP. Data are represented as means ± SEM. $^{**}p < 0.01$, following unpaired t-tests, number of cultures = 6 for control and 3 for *TSC2* neurons. Also see Table 1.

Fig S4

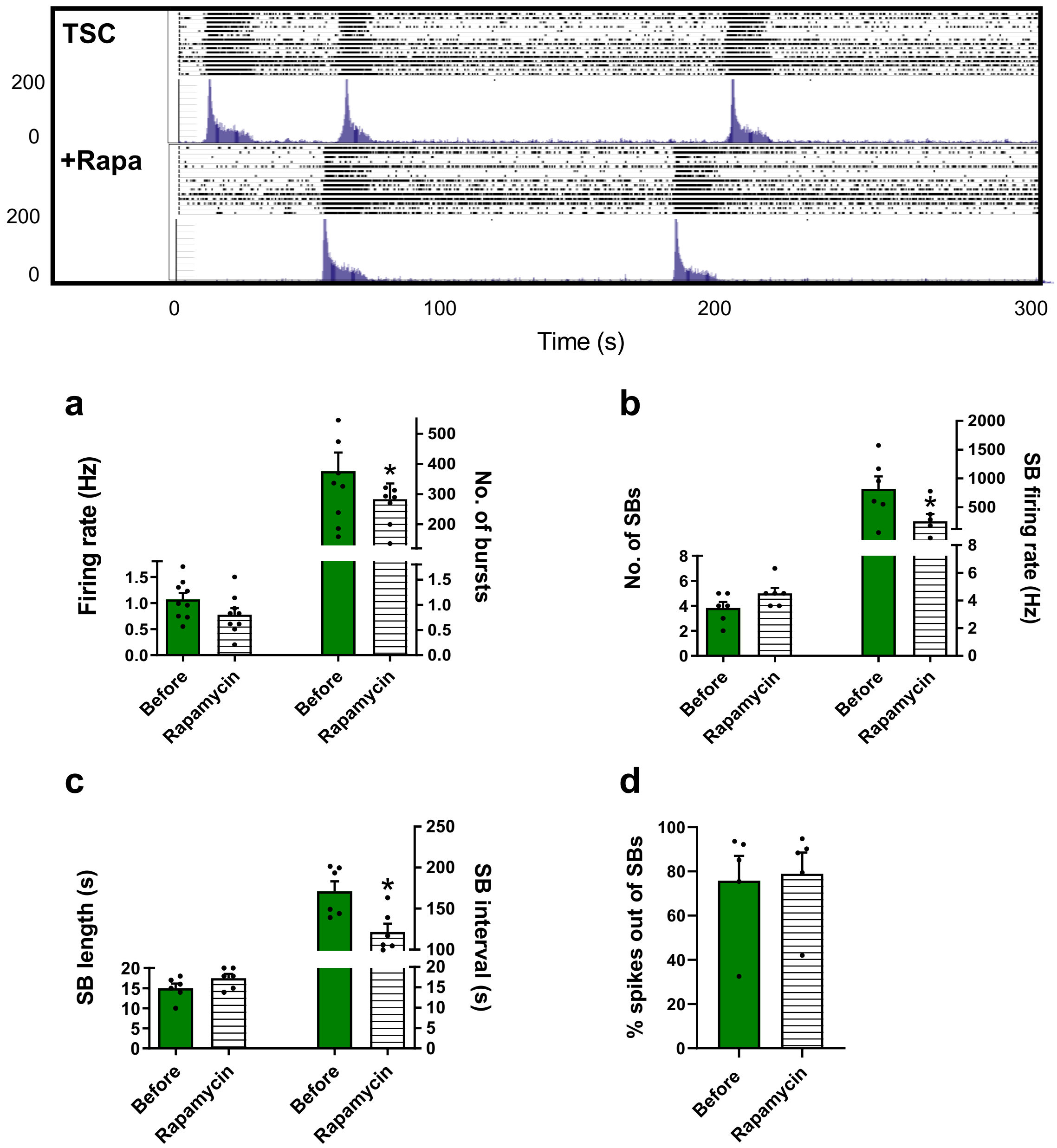

Effects of acute treatment (24 hours) with rapamycin (10 nM) on control neuronal activity. Raster (upper panels) and ASDR plots (lower panels) of a single neuronal MEA culture in the absence and presence of rapamycin (10nM). Vertical scale bar = 200 spikes per 200 ms bin. Changes in (**a**) basal excitability, (**b**) synchronised burst (SB) activity and the number of spikes in individual SBs, (**c**) SB length and interval, and (**d**) the spikes out of SBs in the absence and presence of rapamycin. All plots show means ± SEM. $p > 0.05$ following unpaired t-tests. Number of arrays = 6.

Fig S5

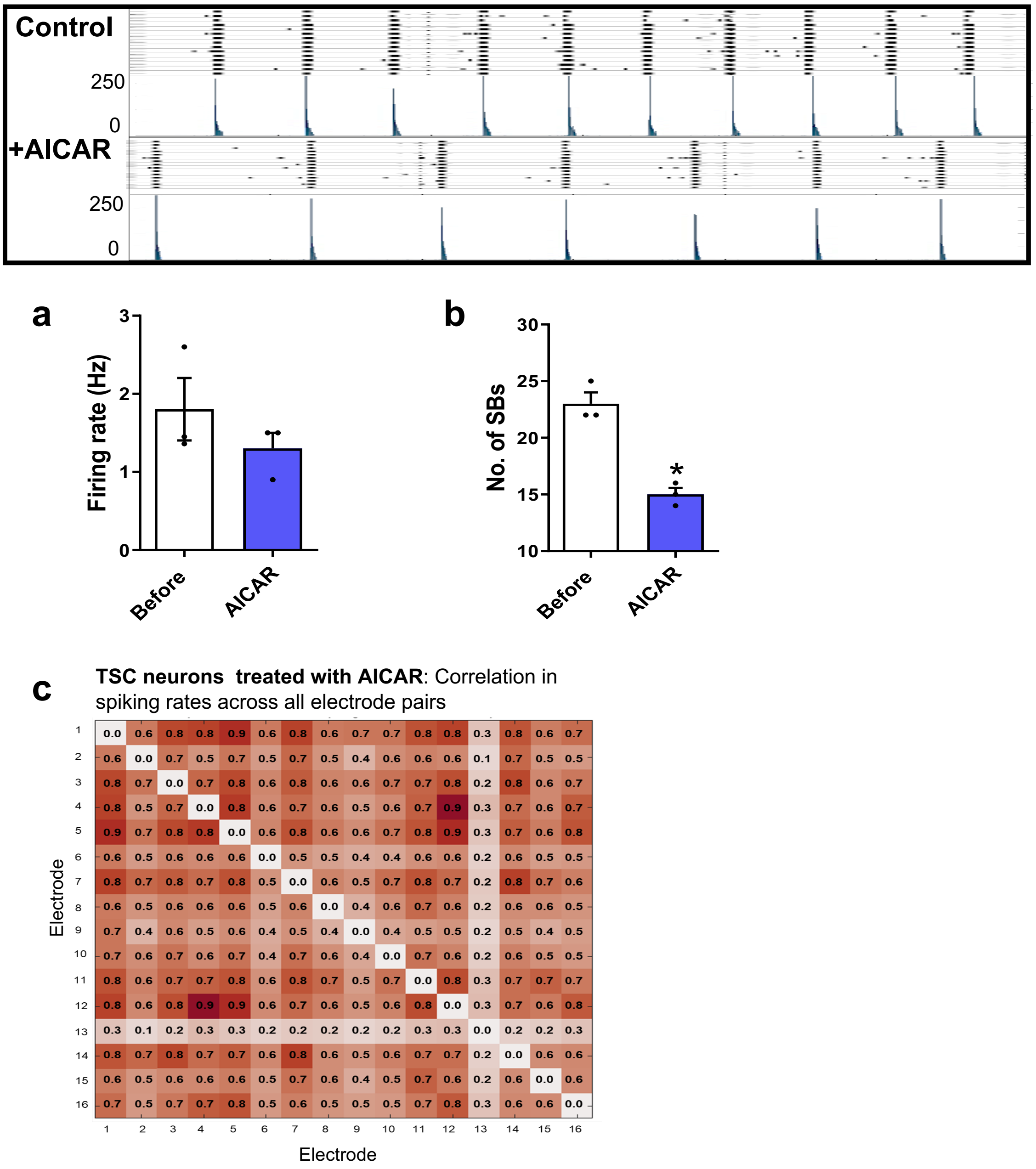

Effects of AICAR on *TSC2* neuronal activity. Raster (upper panel) and ASDR (lower panel) plots of recordings of the same MEA showing 24-hour exposure to AICAR (1 mM). Network activity following 24-hour drug treatment of *TSC2* neurons in the absence and presence of AICAR showing (**a**) spike firing rate (Hz) (**b**) number of synchronised burst (c) Correlation matrices heat map for *TSC2* neurons in the absence and presence of AICAR colours represent the correlation in the firing rates across the indicated electrode. Correlation matrices are calculated for 16 electrodes in control and TCS neurons plated on the MEAs. Values greater than zero represent positive correlation, while values below zero represent negative correlation. All plots show means ± SEM. *$p < 0.05$, following paired t-tests. Number of recorded wells = 7.

Figure S6

A Control **B TSC**

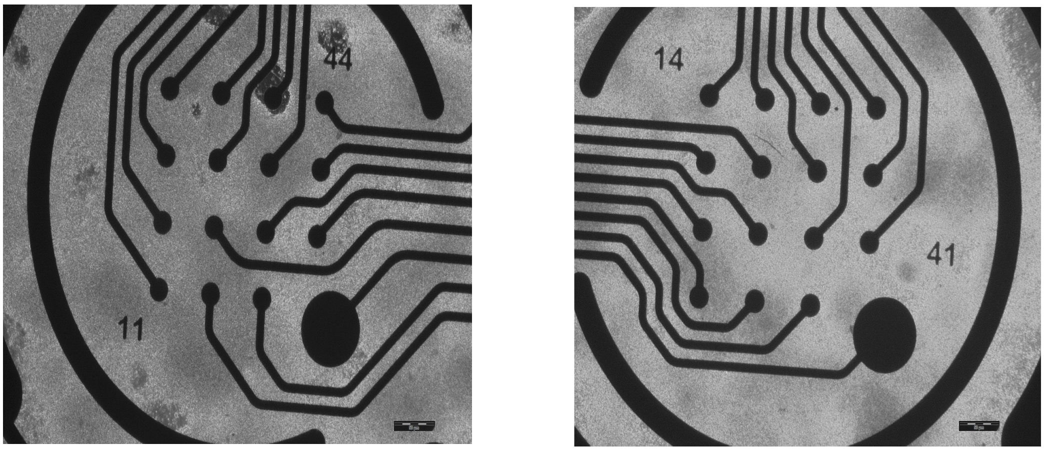

Images of MEA cultures showing typical cell plating of control (A) and *TSC2* (B) neurons.
